# Urokinase receptor promotes ovarian cancer cell dissemination through its 84-95 sequence

**DOI:** 10.18632/oncotarget.1930

**Published:** 2014-05-01

**Authors:** Katia Bifulco, Giuseppina Votta, Vincenzo Ingangi, Gioconda Di Carluccio, Domenica Rea, Simona Losito, Nunzia Montuori, Pia Ragno, Maria Patrizia Stoppelli, Claudio Arra, Maria Vincenza Carriero

**Affiliations:** ^1^ Department of Experimental Oncology Unit, IRCCS Istituto Nazionale Tumori “Fondazione G. Pascale”, Naples, Italy; ^2^ Department of Experimental Pathology Unit, IRCCS Istituto Nazionale Tumori “Fondazione G. Pascale”, Naples, Italy; ^3^ Institute of Genetics and Biophysics “Adriano Buzzati-Traverso”, National Research Council, Naples, Italy; ^4^ Department of Translational Medical Sciences,“Federico II” University, Naples, Italy; ^5^ Department of Chemistry and Biology, University of Salerno, Fisciano (Salerno), Italy

**Keywords:** Urokinase Receptor, Ovarian cancer, Cell Invasion

## Abstract

The clinical relevance of the urokinase receptor (uPAR) as a prognostic marker in ovarian cancer is well documented. We have shown that the uPAR sequence corresponding to 84-95 residues, linking D1 and D2 domains (uPAR_84-95_), drives cell migration and angiogenesis in a protease-independent manner. This study is aimed at defining the contribution of uPAR_84-95_ sequence to invasion of ovarian cancer cells. Now, we provide evidence that the ability of uPAR-expressing ovarian cancer cells to cross extra-cellular matrix and mesothelial monolayers is prevented by specific inhibitors of PAR_84-95_ sequence. To specifically investigate uPAR_84-95_ function, uPAR-negative CHO-K1 cells were stably transfected with cDNAs coding for uPAR D2 and D3 regions and exposing (uPARD2D3) or lacking (uPARΔD2D3) the 84–95 sequence. CHO-K1/D2D3 cells were able to cross matrigel, mesothelial and endothelial monolayers more efficiently than CHO-K1/ΔD2D3 cells, which behave as CHO-K1 control cells. When orthotopically implanted in nude mice, tumor nodules generated by CHO-K1/D2D3 cells spreading to peritoneal cavity were more numerous as compared to CHO-K1/ΔD2D3 cells. Ovarian tumor size and intra-tumoral microvessel density were significantly reduced in the absence of uPAR_84-95_. Our results indicate that cell associated uPAR promotes growth and abdominal dissemination of ovarian cancer cells mainly through its uPAR_84-95_ sequence.

## INTRODUCTION

Epithelial ovarian carcinoma (EOC) is a highly lethal tumor due to its propensity to form widespread peritoneal implants throughout the abdominal cavity. The majority of patients with EOC are not diagnosed until the disease is in advanced stages, which consequently results in a poor outcome. EOC are highly vascularized but the cellular processes that lead to their interactions with endothelium and subsequent invasion through endothelial environment are poorly understood. The metastatic potential of tumor cells depends on many factors, including the ability to cross physical barriers and to interact with the microenvironment at the metastatic site, in turn promoting cell adhesion, growth, survival, angiogenesis, and invasion [1-4]. In particular, ovarian cancer cells detach from the primary tumor and float in the ascitic fluid as single cells or multicellular, chemoresistant spheroids which spread throughout the peritoneal cavity where they adhere and invade through the mesothelium. Therefore, understanding the molecular mechanisms by which ovarian cancer cells adhere to the mesothelium and/or transmigrate through microvessels is a prerequisite to the development of new prognostic tools as well as strategies to limit intra-abdominal tumor dissemination.

The urokinase receptor (uPAR) is emerging as a cell surface-associated molecule relevant to cancer invasion and metastasis. First of all, the clinical relevance of uPAR as a prognostic marker, when measured in tumor tissues and/or plasma, has been demonstrated in various cancer diseases [5]. In EOC, the expression of uPAR is associated with disease progression, indicating that uPAR is a useful marker for EOC prognosis as well as a promising therapeutic target [6]. The uPAR has been reported to be overexpressed by cancer epithelial cells of 92% of ovarian cancer patients whereas it is absent or slightly expressed in normal ovarian surface epithelium [7]. By focusing proteolytic activity of its ligand urokinase plasminogen activator (uPA) on tumour and host cell surface, uPAR contributes to increased vascular permeability and inflammatory ovarian cancer microenvironment [8]. However, to date, the exact mechanistic role of uPAR in ovarian cancer progression and development of peritoneal implants has not been yet elucidated.

The uPAR is a surface receptor consisting in three domains (D1, D2, and D3), anchored to the cell surface through a carboxy-terminal glycosyl-phosphatidyl-inositol anchor [9]. Full uPAR or fragments thereof (deriving from cleavages at protease-sensitive regions of the receptor on tumor cell membranes) may be released in soluble forms in plasma and/or urine. High levels of urinary D1 fragment were found in mice carrying a tumour displaying cleaved uPAR on the cell surface, but little or no D1 was found in the urine from mice carrying a tumour with full-length uPAR, indicating that uPAR fragments in the urine originate from the human tumour [10]. In humans, a high concentration of plasma D1 is an independent preoperative marker of poor prognosis; also, elevated plasma levels of soluble uPAR and derived fragments (D1D2D3, D2D3), together with CA125 marker, discriminate between malignant and benign ovarian tumors [11]. Confirmatory evidence of the crucial role of uPAR in tumor dissemination stems from experiments with anti-uPAR ATN-658 antibody which reduces metastases in an ovarian cancer xenograft model [7].

Although the relationship between uPAR function, ovarian cancer pathogenesis and development of peritoneal implants remains largely elusive, the emerging role of uPAR in prognosis, diagnosis and anti-cancer therapy deserves attention. When expressed on cell surface, uPAR promotes cell-associated proteolysis by binding to uPA, which locally converts plasminogen into active plasmin, thus favoring tissue invasion and metastasis [12]. Furthermore, ligand-engaged uPAR also acts as a potent regulator of tumor cell migration and matrix attachment, independently of its catalytic activity [13-14]. In the past years, we and others have shown that signaling occurs through the assembly of uPAR in composite regulatory units with extracellular matrix (ECM) proteins such as vitronectin, and with transmembrane receptors like the G protein-coupled formyl-peptide receptors (FPRs) as well as integrins [15-23].

The X-ray studies have shown that the three domains pack together into a concave structure that binds uPA, and that the domain boundary between uPAR D1-D2 is more flexible than the D2-D3 domain boundary [24-26]. The linker region between D1 and D2 (uPAR_84-95_) is a protease sensitive region which retains chemotactic activity [17, 27]. Plasmin generated by uPA or uPA itself can cleave intact uPAR (D1D2D3), releasing D1. The remaining GPI-anchored D2D3 can be left on cell surface or be secreted in the extracellular milieu following cleavage of the anchor [28].

A crucial signaling region of membrane-associated uPAR is the minimal active ^88^Ser-Arg-Ser-Arg-Tyr^92^ sequence able to trigger cell migration and angiogenesis *in vitro* and *in vivo*. A synthetic peptide SRSRY retains the pro-migratory ability of full uPAR [19, 29]. Mechanistically, uPAR_84-95_ sequence promotes cell motility by interacting with G protein coupled FPR which, in turn, triggers vitronectin receptor activation with an inside-outside type of mechanism [19].

Previous work from this laboratory showed that substitution of Ser90 in full length, membrane-associated uPAR, affects the complex uPAR/FPR/vitronectin receptor cross-talk [30]. Interestingly, penta- and tetra-peptides carrying Ser90 substituted with a glutamic acid residue revealed an intrinsic inhibitory activity of uPAR_84-95_-dependent signaling [31-32]. Among these, the N-terminal acetylated and C-terminal amidated Ac-Arg-Glu-Arg-Phe-NH_2_ peptide, namely RERF, potently inhibits *in vitro* and *in vivo* cell migration and invasion of human fibrosarcoma HT1080 cells without affecting cell proliferation. Cell exposure to RERF results in the inhibition of both uPAR/FPR and uPAR/vitronectin receptor interactions. These effects are supported by the identification of FPR as the main binding site of RERF and αv integrin subunit as a low affinity binding site (Kdsapp, 10^−17^M and 10^−13^M, respectively) [32]. More recently, we have documented that RERF prevents not only uPAR_84-95_-induced but also VEGF-induced angiogenesis *in vitro* and *in vivo* [33].

To date, the mechanistic role of uPARD2D3 in ovarian cancer progression and development of peritoneal implants has not been completely understood. In the present study, our aim was to investigate the contribution of membrane-associated uPAR_84-95_ to invasion of ovarian cancer cells *in vitro* and *in vivo*. Using human ovarian carcinoma SKOV-3 cells expressing uPAR, hamster ovarian CHO-K1 cells lacking uPAR and stably transfected with cDNAs coding for GPI-anchored, truncated forms of uPARs lacking the N-terminal D1 domain and exposing (uPARD2D3) or lacking (uPARΔD2D3) the 84–95 sequence, we investigated whether the presence, or the deletion of uPAR_84–95_ plays a role in the growth and intra-abdominal dissemination of ovarian cells orthotopically implanted in nude mice.

## RESULTS

### Requirement of the uPAR_84-95_ sequence for ovarian cancer cell migration

**Figure 1:**
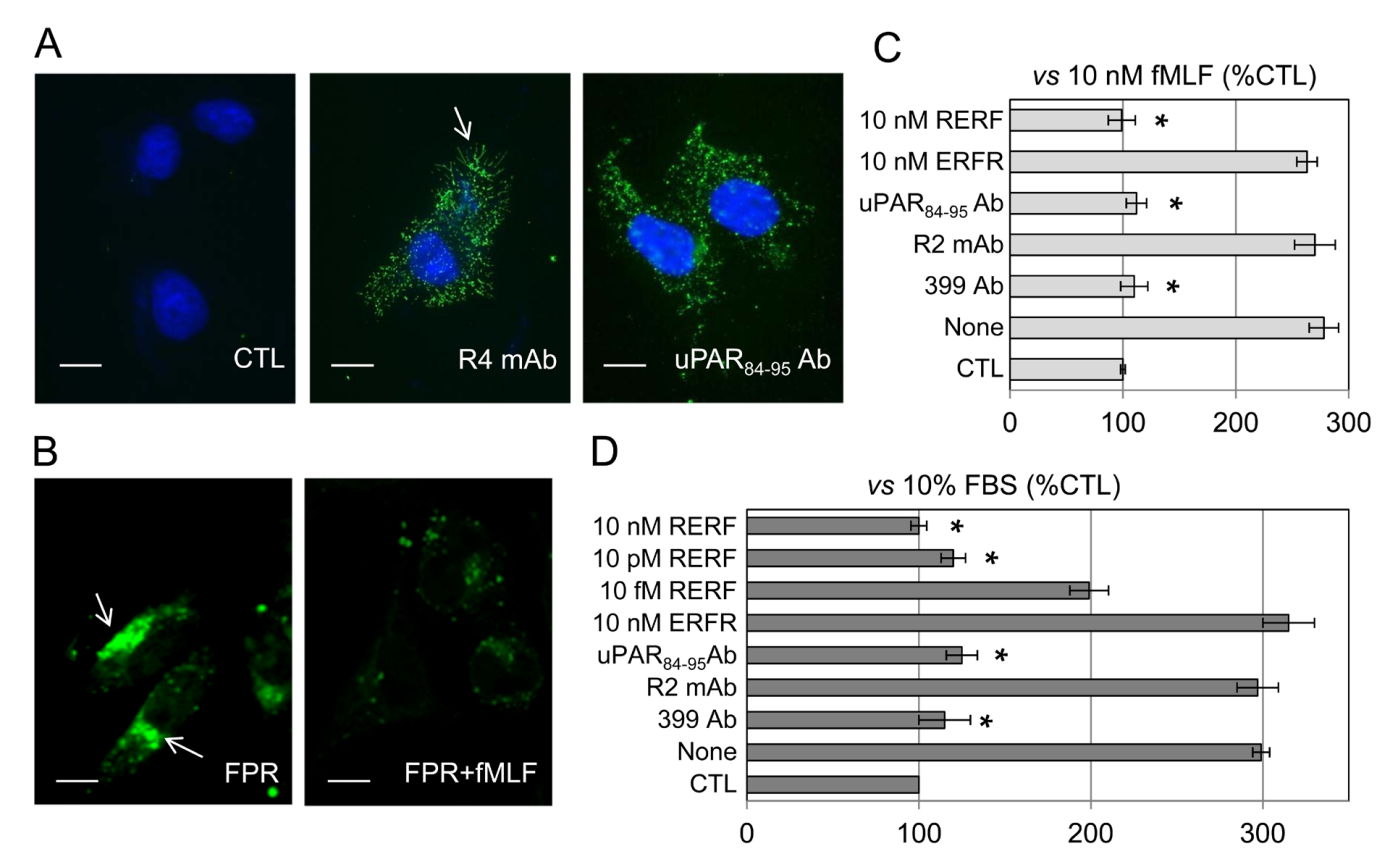
Inhibition of SKOV-3 cell migration by anti-uPAR and RERF peptide A: Representative images of human ovarian carcinoma SKOV-3 cells incubated with PBS (CTL), 2 μg/mL R4 anti-uPAR monoclonal antibody or rabbit anti-uPAR_84-95_polyclonal antibody overnight at 4°C, exposed to Alexa Fluor 488-conjugated F(ab')2 fragment of rabbit anti-mouse IgG or Alexa Fluor 488 goat anti-rabbit IgG for 40 minutes at 23°C and visualized by a fluorescence inverted microscope Nuclei were stained blue with DAPI. Arrow indicates R4-stained uPARs on membrane protrusions. Scale bar: 10 μm. Original magnification: 1000 x. **B**: Representative images of SKOV-3 cells incubated with diluents (FPR) or 100 nM fMLF (FPR+fMLF) for 30 min at 37°C, exposed to 10 nM N-formyl-Nle-or Leu-Phe-Nle-Tyr-Lys-fluorescein for additional 30 min at 37°C and then visualized using a Zeiss 510 META LSM microscope. Arrows indicate the intra-cytoplasmic green fluorescent spots. Scale bar: 10 μm. Original magnifications: 630x. **C-D**: Cell migration of SKOV-3 cells allowed to migrate in Boyden chambers for 4 hrs at 37°C using 10 nM fMLF (C) or 10% FBS (D) as chemoattractants, in the presence or the absence of diluents (none), 2 μg/mL 399 anti-uPAR polyclonal antibody, 2 μg/mL anti-uPAR_84-95_ polyclonal antibody, 2 μg/mL R2 anti-uPAR monoclonal antibody, or the indicated peptides. For quantitative analysis of cell migration, the basal value assessed in the absence of chemoattractant (CTL) was taken as 100% and all values were reported relative to that. Data are the means ± SD of two independent experiments, performed in triplicate. *Statistical significance calculated against the positive control (None) with p < 0.001.

This work was aimed to identify the specific contribution of the uPAR_84-95_ region in promoting dissemination of ovarian cancer cells bearing GPI-anchored uPAR. As a first approach, we employed the invasive human ovarian cancer SKOV-3 cells [34] expressing a considerable amount of surface uPAR. As shown in Figure 1A, R4 anti-uPAR monoclonal antibody appears to localize on overall cell surface, mostly on membrane protrusions (arrow), whereas staining with anti-uPAR_84-95_ is equally distributed on the whole cell surface. Upon uPA engagement, uPAR undergoes conformational changes favoring the exposure of the uPAR_84-95_ sequence which directly interacts with fMLF receptors and, in turn, triggers vitronectin receptor activation [19, 30]. A likely explanation is that anti-uPAR_84-95_ Abs recognize a subset of R4-stained receptors, possibly those not involved in proximal interactions, while R4 mAb detects uPAR D3 regardless of its engagement with ligand and proximal receptors (eg FPR).We and others have previously documented that uPAR binds to FPR through its Ser^88^-Arg-Ser-Arg-Tyr^92^ sequence, thus promoting FPR internalization and cell migration [18-19]. Thus, we assessed whether SKOV-3 cells express FPR and whether agonist-dependent FPR internalization does occur following exposure to 10 nM N-formyl-Nle-Leu-Phe-Nle-Tyr-Lys-fluorescein for 30 min at 37°C. FPR, probed by its fluorescent agonist and observed by a confocal microscopy, appeared as intra-cytoplasmic green fluorescent spots (arrows) which disappeared in cells pre-incubated with an excess of non-fluorescent fMLF (Figure 1B). Then, uPAR ability to trigger FPR activation was investigated by cell migration assays using fMLF as chemoattractant. We found that 10 nM fMLF elicits a considerable SKOV-3 cell migration, reaching 278% of the basal cell migration that was fully prevented by 399 anti-uPAR (American Diagnostica) as well as by anti-uPAR_84-95_ polyclonal antibodies but not by the R2 anti-uPAR monoclonal antibody which recognizes an epitope located on the D3 domain (Figure 1C). These findings indicate that, as previously described for other cell lines [18-20, 29, 31-32], uPAR activates fMLF-induced and FPR-mediated migration of SKOV-3 cells through its uPAR_84-95_ sequence. We have previously shown that the Ac-Arg-Glu-Arg- Phe-NH_2_ (RERF) peptide, corresponding to the minimal uPAR region interacting with FPR and carrying the Ser90E substitution, inhibits cell migration by preventing uPAR_84-95_/FPR interaction [32]. We found that, unlike the scrambled control peptide ERFR, 10 nM RERF (corresponding to a 8.7675 ng/mL concentration) fully abrogated fMLF-induced SKOV-3 cell migration (Figure 1C). To evaluate the role of uPAR_84-95_ in a system more representative of the *in vivo* context, SKOV-3 cells were tested for their ability to migrate toward serum. Not surprisingly, 10% FBS elicited a considerable cell migration, reaching 299% of the basal cell migration. Both 399 anti-uPAR and anti-uPAR_84-95_ polyclonal antibodies reduced cell migration almost to basal levels, whereas the R2 monoclonal antibody did not exert such effect, supporting a crucial role of uPAR in SKOV-3 cell migration (Figure 1D). According to the previously reported dose-dependent inhibitory effect [32], RERF reduced FBS-dependent cell migration in a dose-dependent manner. In particular, 10 fM and 10 pM RERF reduced cell migration by 35%, and 60%, respectively (Figure 1D). These findings confirm the relevance of uPAR and highlight the role of the uPAR_84-95_ sequence to promote ovarian cancer cell migration.

### Requirement of the uPAR_84-95_ sequence to SKOV-3 ovarian cancer cell invasion

**Figure 2:**
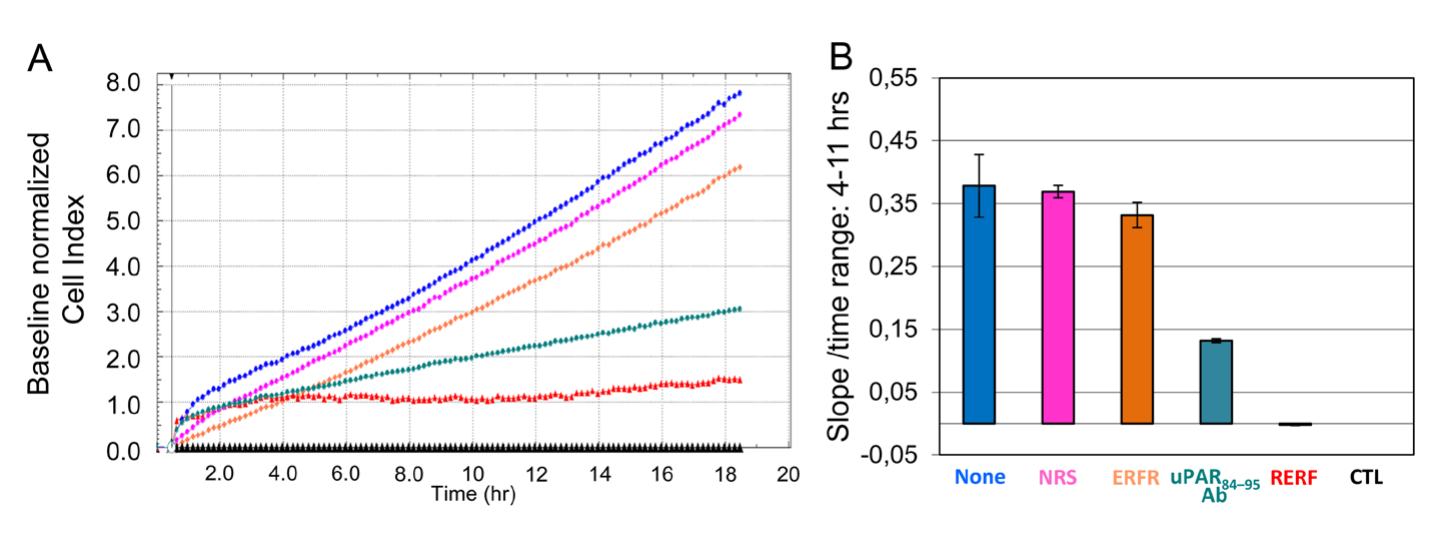
Relevance of uPAR_84-95_ sequence to ECM invasion by SKOV-3 cells Matrigel invasion of SKOV-3 cells was monitored by the xCELLigence system. SKOV-3 cells were seeded on polymerized matrigel in CIM-16-well plates and allowed to invade matrigel for 18 hrs. Lower chambers were filled with DMEM as control (CTL) or growth medium plus/minus 2 μg/mL normal rabbit serum (NRS), 2 μg/mL rabbit anti-uPAR_84-95_ polyclonal antibody, 10 nM of the indicated peptides or diluents (None). **A.** Invasion was monitored in real-time for 18 hrs as changes in Cell Index. Cell Index values were normalized immediately after SKOV-3 cell addition and the impedance values of cells invading matrigel toward DMEM alone (CTL) were equated to 0 (baseline). **B.** Slopes represent the change rate of Cell Indexes generated in a 4-11 hrs time frame.

Since cell motility is a prerequisite for the acquisition of an invasive phenotype, we explored the ability of SKOV-3 cells to invade basement membranes and mesothelial monolayers by the aid of uPAR_84-95_ sequence. The ability of SKOV-3 cells to invade matrigel, a reconstituted basement membrane, was assessed using the xCELLigence RTCA technology in which impedance changes are caused by the presence of cells. SKOV-3 cells were seeded on polymerized matrigel and lower chambers were filled with DMEM or growth medium with or without 2 μg/mL normal rabbit serum (NRS), 2 μg/mL anti-uPAR_84-95_ polyclonal antibody, or 10 nM of the indicated peptides. Matrigel invasion was monitored in real-time for 18 hrs as Cell Index changes due to the adhesion of invading cells to microelectrodes. Cell Index values were normalized immediately after SKOV-3 cell addition and the impedance values of samples without chemoattractant (CTL) were equated to 0 (baseline). As expected, basal invasion of SKOV-3 cells did not change significantly neither in the presence of NRS nor with the scrambled control peptide ERFR. Conversely, anti-uPAR_84-95_ polyclonal antibody or 10 nM RERF reduced index values of cells invading toward serum by 50% and 86%, respectively (Figure 2A). These differences appeared more evident when slopes, which represent the rate of change of the Cell Index, were generated in the range of 4 to 11 hrs (Figure 2B).

### Requirement of the uPAR_84-95_ sequence to SKOV-3 ovarian cancer cell mesothelial invasion

**Figure 3:**
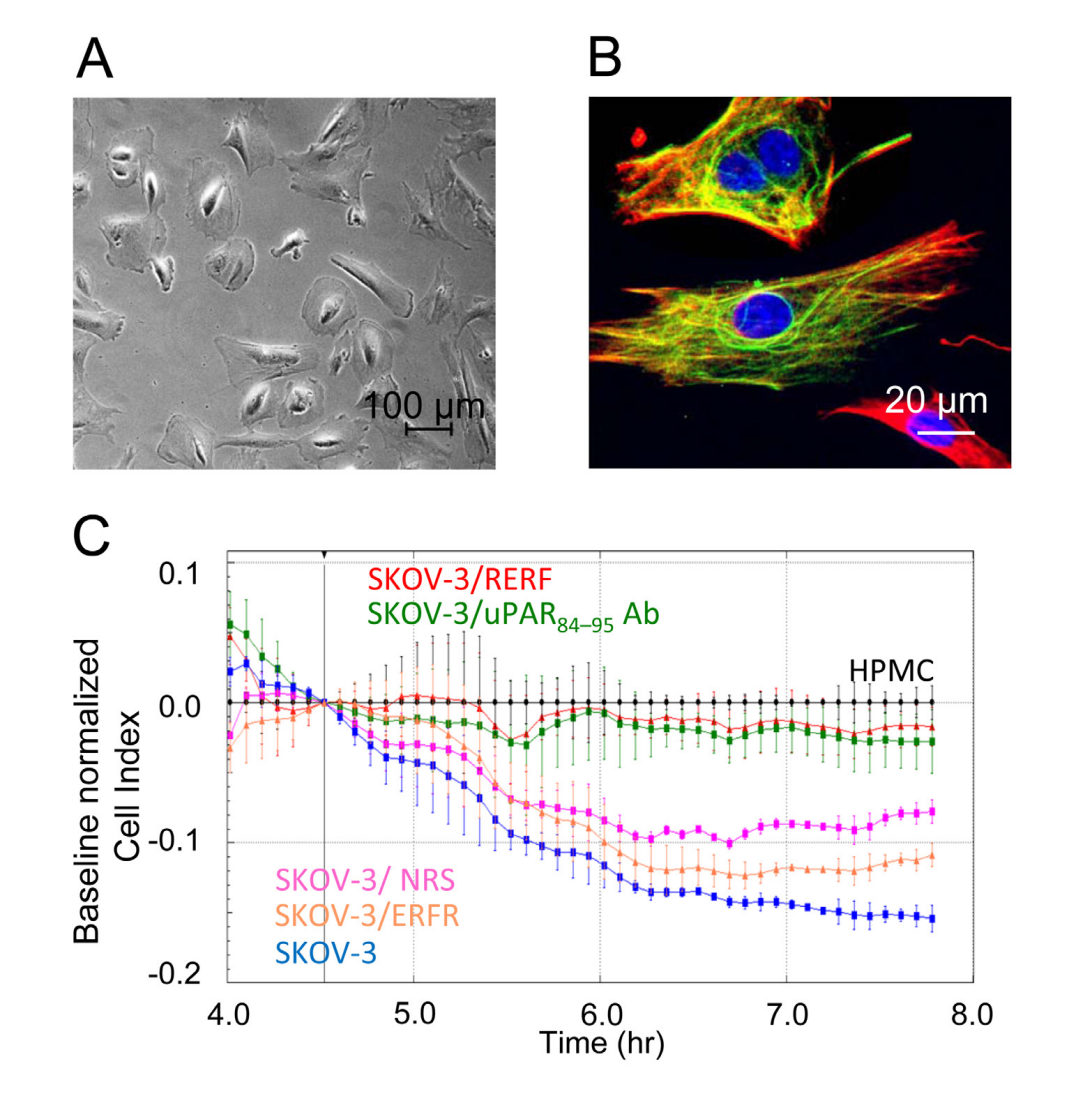
Relevance of uPAR_84-95_ sequence to invasion of HPMC monolayer by SKOV-3 cells HPMCs were purified from human omental specimens and cultured as described in the Materials and Methods section. **A-B**. Representative images of HPMCs analyzed by phase contrast microscopy **(A)** or stained for cytokeratin 8/18 (green) and vimentin (red) **(B)**. Nuclei were stained blue with DAPI. Scale bar: 20 μm. Original magnification: 1000 x. C. 5×10^3^ HPMCs were seeded in E-16-well plates in growth medium and allowed to adhere for 4 hrs to form a confluent monolayer. Then, SKOV-3 cells (2×10^4^ cells/well) were seeded in 10% FBS plus/minus 10 nM of indicated peptides, 2 μg/mL normal rabbit serum (NRS) or 2 μg/mL anti-uPAR_84-95_ polyclonal antibody. SKOV-3 cells invasion was monitored in real-time as changes in Cell Index due to breaking of monolayer integrity. Cell Index values were normalized immediately after SKOV-3 cell addition and the impedance values of HPMCs (black line) alone were equated to 0 (baseline). Data represent mean ± SD from a quadruplicate experiment representative of 2 replicates.

SKOV-3 cell ability to adhere to and cross peritoneum was tested by employing HPMCs purified from human omental specimens according to Stylianou et al. [35]. HPMC cultured cells were identified as 75% pure mesothelial cells by their cobblestone appearance at semi-confluence (Figure 3A), as well as by positive staining for cytokeratin 8/18 (green) and vimentin (red) (Figure 3B) and negative staining for von Willebrand factor VIII related antigen (not shown). To obtain a monolayer, 5×10^3^ HPMCs, employed between the second to the third passage, were seeded on E-16-well plates and allowed to adhere for 4 hrs at 37°C, 5% CO_2_. Then, SKOV-3 cells (2×10^4^ cells/well) were seeded onto the monolayer in the presence of 10% FBS with or without 2 μg/mL of the indicated antibody or 10 nM of the indicated peptides. The reduction of impedance values, due to invading cells that interrupt monolayer integrity was monitored in real-time for subsequent 4 hrs. Addition of SKOV-3 cells lead to rupture of monolayer integrity which was mostly prevented by anti-uPAR_84-95_ polyclonal antibody and by 10 nM RERF (Figure 3C). These findings suggest that uPAR supports SKOV-3 cell invasion and that this ability resides mainly in the uPAR_84-95_ region.

### Generation of uPAR_84-95_ expressing CHO-K1 ovarian cells

**Figure 4:**
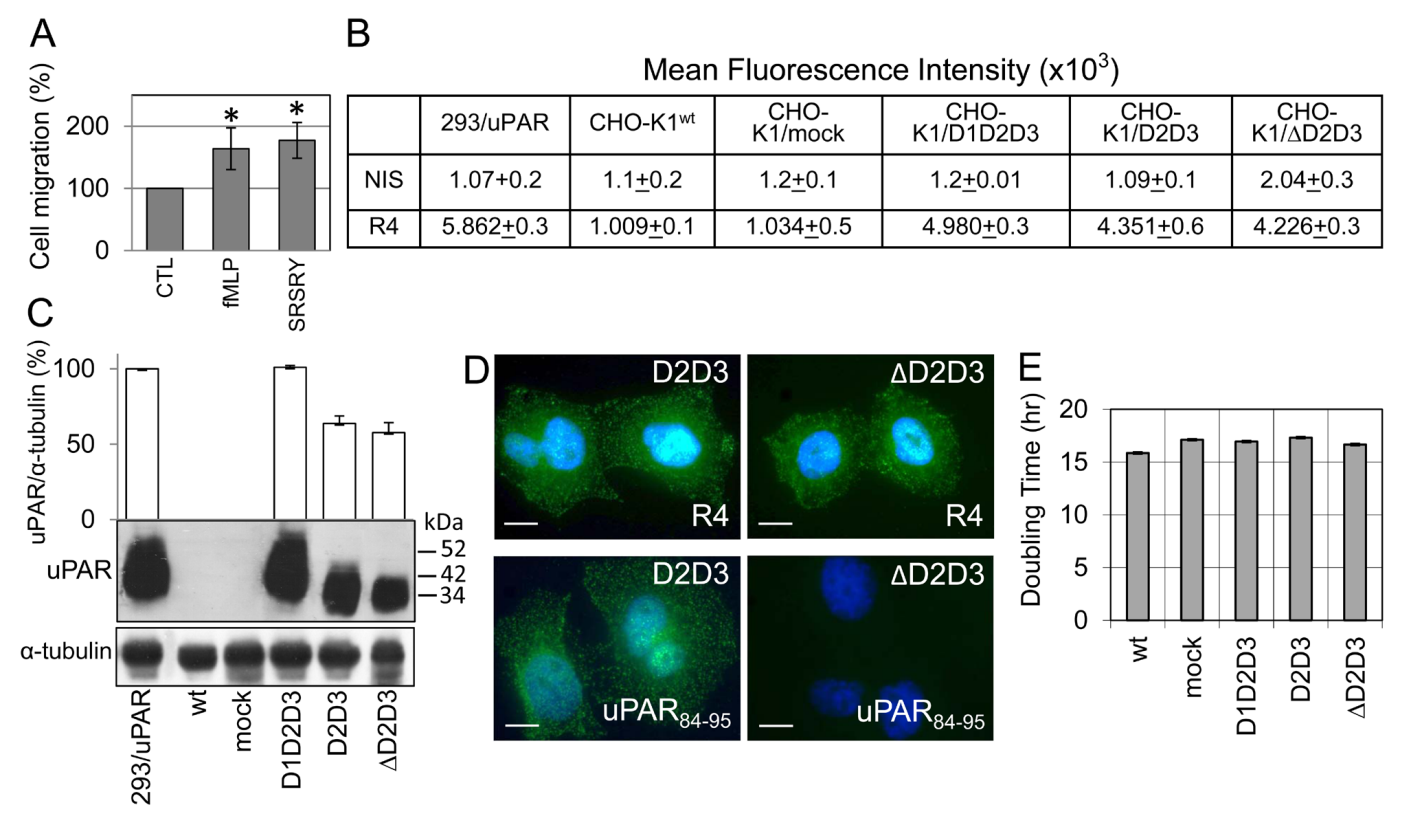
Generation of CHO-K1 ovarian stable transfectants either lacking or expressing the uPAR sequence CHO-K1 cells were stably transfected with pcDNA3 empty vector (mock) or pcDNA3 loaded with wild type uPAR (D1D2D3), D2D3 or ΔD2D3 cDNAs **A**: Wild type CHO-K1 cells were tested for directional migration in Boyden chambers for 4 hrs at 37°C toward 10 nM SRSRY or 10 nM fMLF. For quantitative analysis of cell migration, the basal value assessed in the absence of chemoattractant (CTL) was taken as 100% and all values were reported relative to that. Data are the means ± SD of two independent experiments, performed in triplicate. *Statistical significance calculated against CTL with p <0.001. **B**: Cytofluorimetric analysis of parental (wt) and stably transfected CHO-K1 cells with R4 anti-uPAR monoclonal antibody or non-immune serum (NIS). HEK-293/uPAR cells were employed as a positive control. Data represent mean ± SD from a duplicate experiment representative of 3 replicates. C: Whole cell lysates (25 μg/sample) from wild type (wt) or transfected CHO-K1 cells were resolved on a 10% SDS-PAGE followed by Western blotting with R4 anti-uPAR monoclonal antibody or anti-α tubulin polyclonal antibody as loading control. 25 μg of HEK-293/uPAR cell lysate were employed as a positive control. The enclosed bar graph shows the average quantification of the uPAR/α-tubulin content expressed as a percentage of the HEK-293/uPAR sample, from 2 independent experiments. **D**: Representative images of CHO-K1/D2D3 and CHO-K1/ΔD2D3 cells immune-stained with R4 anti-uPAR monoclonal antibody or rabbit anti-uPAR_84-95_ polyclonal antibody. Nuclei were stained blue with DAPI. Scale bar: 10 μm. Original magnification: 1000 x. **E**: Cell proliferation of the indicated cells assessed by monitoring impedance by RTCA xCELLigence system. The reported doubling times were calculated from the cell growth curves, during exponential growth. Data represent mean ± SD from a quadruplicate experiment representative of 3 replicates.

To study the relevance of uPAR_84–95_ sequence in an uPAR-negative cellular background, further experiments were performed using uPAR-negative CHO-K1 cells which have been documented to retain metastatic capability when injected in immunodeficient mice [36-37], probably due to their high production of matrix-degrading enzymes [38]. First of all, we assessed whether wild type CHO-K1 cells respond to FPR agonists by directional cell migration. As shown in Figure 4A, both fMLF and SRSRY which are documented to target FPRs [17, 19], elicited migration of CHO-K1 cells at 10 nM, reaching 164% and 177% of the basal cell migration, respectively. Therefore, wild type and truncated human uPAR forms were stably expressed in CHO-K1 cells and transfectants were subjected to functional studies. CHO-K1 cells, were stably transfected with pcDNA3 empty vector (mock) or pcDNA3 carrying cDNAs encoding wild type, full length uPAR (D1D2D3), or its truncated forms lacking D1 domain and containing the uPAR_84–95_ sequence (D2D3), or lacking both D1 domain and uPAR_84–95_ sequence (ΔD2D3). CHO-K1 stable transfectants were analysed by cytofluorimetry for uPAR expression level on cell surface, using R4 anti-uPAR monoclonal antibody, recognizing the D3 domain. Mean fluorescence intensity values associated to wild type CHO-K1 or CHO-K1/mock cells were comparable to those obtained in the presence of non-immune serum, confirming the absence of uPAR. Instead, an appreciable amount of uPAR was found on CHO-K1/D1D2D3 cells as compared to human uPAR overexpressing HEK-293/uPAR cells [39], and a moderate but comparable expression of truncated uPAR forms on CHO-K1/D2D3 and CHO-K1/ΔD2D3 cell surfaces (Figure 4B). In Western blots, wild type CHO-K1 and CHO-K1/mock cell lysates did not react to R4 anti-uPAR monoclonal antibody, whereas samples from CHO-K1/D1D2D3 exhibited a single band co-migrating with uPAR from HEK-293/uPAR control cells. Unlike CHO-K1/D1D2D3 cells, both D2D3 and ΔD2D3 transfectants express truncated uPARs (in a ratio of 1.1 : 1), with a molecular weight of about 35 kDa, compatible with the cleaved D2D3 fragment of uPAR [18] (Figure 4C). Accordingly, R4 anti-uPAR antibody recognizes uPAR on cell surfaces of both D2D3 and ΔD2D3 transfectants whereas only D2D3 transfected cells were recognized by anti-uPAR_84-95_ antibody (Figure 4D). Further comparison of the transfectants with regard to cell proliferation rate was assessed using the xCELLigence technology. This procedure revealed that all stably transfected CHO-K1 cells exhibit comparable doubling times (16,8 +/−0,5 hrs), calculated from the cell growth curve during exponential growth (Figure 4E). In conclusion, these results show that all transfectants exhibit comparable growth rates and that CHO-K1 cells carrying D2D3 or ΔD2D3 express similar amounts of truncated forms of uPAR on their surface, thus being suitable for functional assays.

### Requirement of the uPAR_84-95_ sequence to CHO-K1 cell migration and matrigel invasion

**Figure 5:**
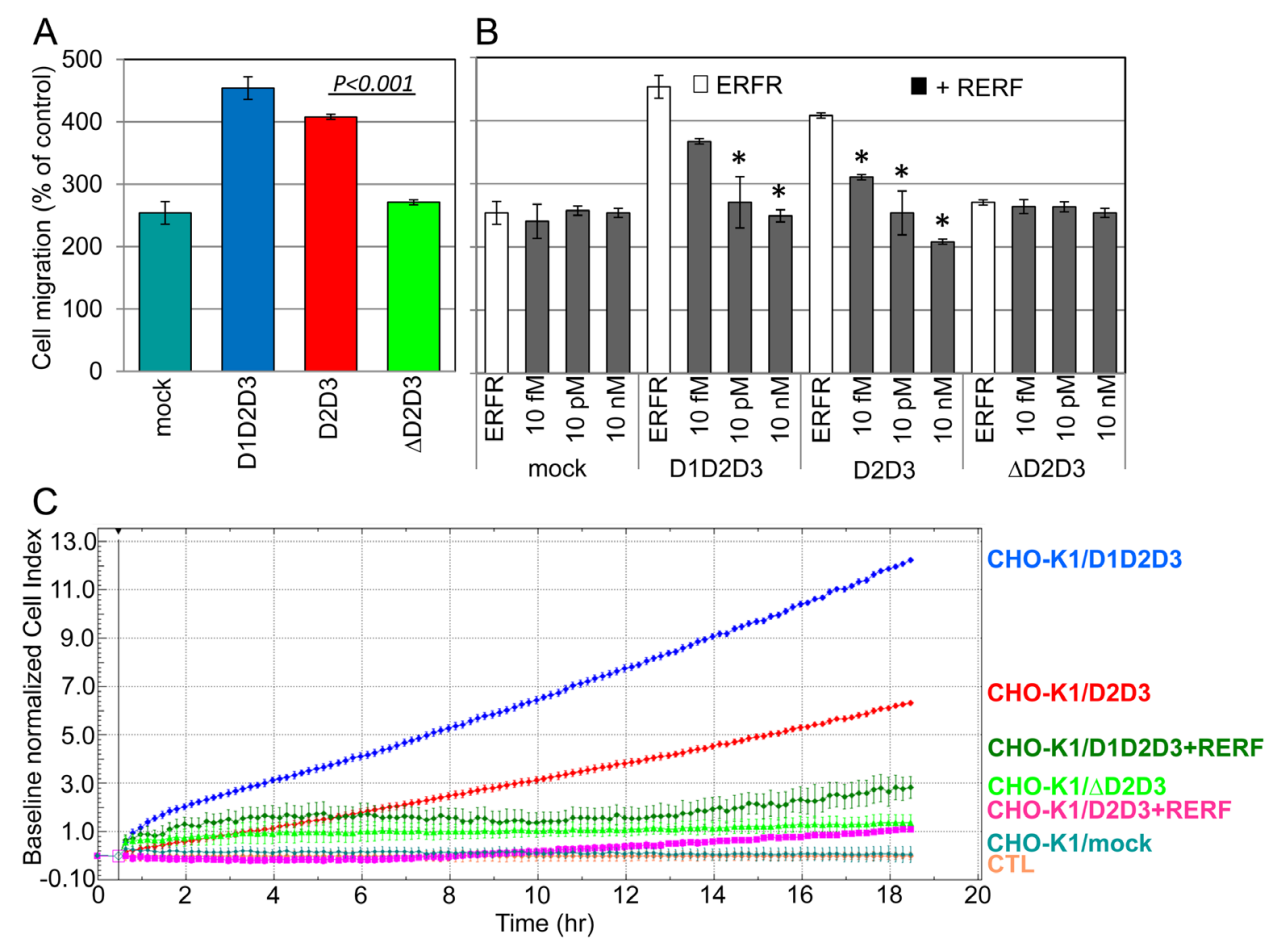
Requirement of uPAR_84-95_ sequence to CHO-K1 cell migration and ECM invasion A: CHO-K1 transfectants were allowed to migrate in Boyden chambers for 4 hrs, toward 10% FBS. For quantitative analysis of cell migration, the basal value assessed in the absence of chemoattractant was taken as 100% and all values were reported relative to that. Data are the means ± SD of two independent experiments, performed in triplicate. **B**: CHO-K1 transfectants cells were allowed to migrate in Boyden chambers for 4 hrs, toward 10% FBS plus 10 nM ERFR or the indicated concentration of RERF. Quantitative analysis of cell migration has reported of the percentage of basal cell migration assessed in the absence of chemoattractant. Data are the means ± SD of two independent experiments, performed in triplicate. *Statistical significance of all samples was calculated against the control (ERFR) with P < 0.001. C: Matrigel invasion of CHO-K1 transfectants was monitored by the xCELLigence system. Cells were seeded on polymerized matrigel in CIM-16-well plates and allowed to invade matrigel in the presence or in the absence of 10 nM RERF. Matrigel invasion was monitored in real-time for 18 hrs as changes in Cell Index. Values were normalized immediately after transfected CHO-K1 cell addition and the impedance values of CHO-K1/mock cells invading matrigel toward F12 alone (CTL) were equated to 0 (baseline). Data represent mean ± SD from a quadruplicate experiment representative of 2 replicates.

CHO-K1 transfectants were first employed to assess the specific contribution of uPAR_84–95_ sequence to cell migration. Although at a different extent, both CHO-K1/D1D2D3 and CHO-K1/D2D3 cells exhibit an appreciable ability to migrate toward FBS (454+/−18% and 408+/−4% of the basal cell migration, respectively), higher than CHO-K1/mock cells (254+/−20%) (Figure 5A). Interestingly, the extent of migration of CHO-K1/mock cells was almost comparable to that of CHO-K1/ΔD2D3 cells lacking D1 domain and the uPAR_84–95_ sequence (254+/−20% and 271+/−6%, respectively). This finding indicates that D1 domain is dispensable for ovarian cell migration, provided the uPAR_84-95_ chemotactic sequence is in place. Unlike control ERFR peptide, RERF reduced migration only of transfectants bearing the uPAR_84–95_ sequence (CHO-K1/D1D2D3 and CHO-K1/D2D3), whereas it did not affect CHO-K1/mock and CHO-K1/ΔD2D3 cell motility (Figure 5B). These findings indicate that uPAR_84-95_ sequence is definitely relevant to ovarian cell motility. Next, the ability of transfected CHO-K1 cells to invade matrigel was assessed using the xCELLigence RTCA technology as above described. Cells were seeded on polymerized matrigel using CIM-16-well plates. Lower chambers were filled with F12 with or without serum, plus/minus 10 nM RERF. Invasion was monitored in real-time for 18 hrs. We found that CHO-K1/D1D2D3 cells exhibit the highest ability to cross matrigel in the presence of serum as compared to both D1-lacking, truncated forms. In particular, CHO-K1/D1D2D3 cells cross matrigel to a double extent than CHO-K1/D2D3 (Figure 5C). The functional differences observed between full length and truncated uPAR forms are likely due to full length uPAR engagement by uPA contained in FBS. In keeping with the previously reported ability of RERF to reduce SKOV-3 cell invasion, the addition of RERF reduced Cell Index values of CHO-K1/D1D2D3 and CHO-K1/D2D3 cells by 75% and 83%, respectively, as assessed after 18 hrs invasion. These findings confirm that the occurrence of membrane-associated uPAR_84–95_ sequence is a pre-requisite for ovarian cells to acquire a migratory/invasive phenotype.

### Requirement of the uPAR_84-95_ sequence to CHO-K1 invasion of a HPMC or HUVEC monolayers

**Figure 6:**
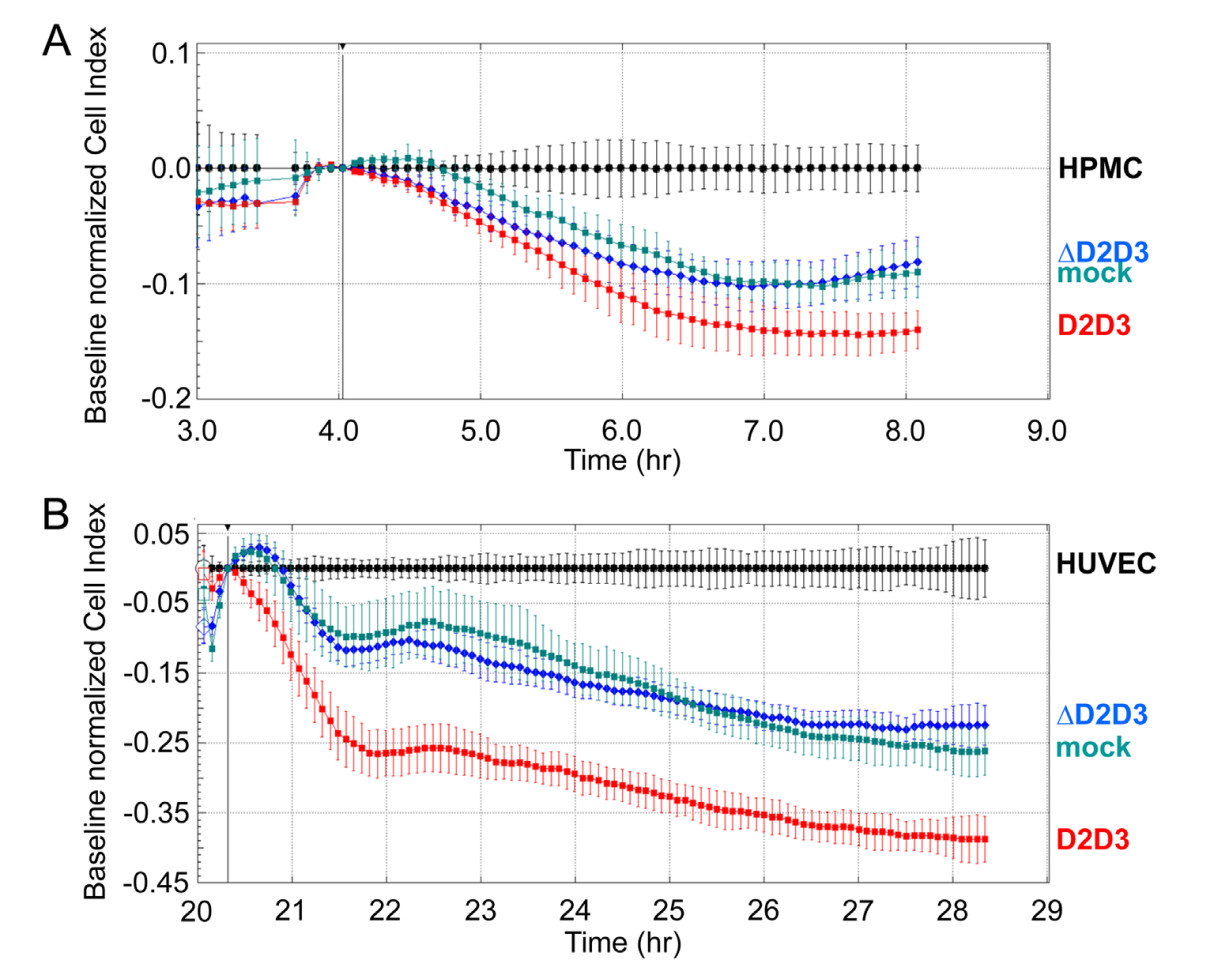
Requirement of uPAR_84-95_ sequence to CHO-K1 invasion of HPMC or HUVEC monolayers 5×10^3^ HPMCs **(A)** or 1×10^4^ or HUVECs **(B)** were seeded in E-16-well plates in growth medium and allowed to adhere for 4 hrs **(A)** or grow for 20 hrs **(B)** to form a confluent monolayer prior to seeding CHO-K1/mock, CHO-K1/D2D3 or CHO-K1/ΔD2D3 cells (2×10^4^ cells/well) in 10% FBS. Monolayer invasion was monitored in real-time as specified in the legend to figure 3. The impedance values of HPMCs **(A)** or HUVECs **(B)** alone were equated to 0 (baseline). Data represent mean ± SD from a quadruplicate experiment representative of 2 replicates.

The role of uPAR_84-95_ in the acquisition of an invasive phenotype was further investigated by analysing the ability of engineered CHO-K1 cells to invade mesothelial or endothelial cell monolayers. Transfected CHO-K1 cells were seeded on a monolayer of HPMCs (Figure 6A) or HUVECs (Figure 6B) using E-16-well plates. As already mentioned above, HPMCs or HUVECs were allowed to adhere or grow until they formed a monolayer for 4 or 20 hrs, respectively, prior to seeding transfected CHO-K1 cells (2×10^4^ cells/well) in the presence of 10% FBS. At this time, reduction of impedance values, due to invading cells that interrupt monolayers were monitored in real-time for 8 hrs. Although to a different extent, all CHO-K1 transfectants were able to invade both mesothelial and endothelial monolayers. According to matrigel invasion data, CHO-K1/D2D3 interrupted mesothelial (Figure 6A) and endothelial (Figure 6B) monolayers more efficiently than CHO-K1/mock and CHO-K1/ΔD2D3 cells. Taken together, these findings highlight the potency of uPAR_84-95_ sequence to endow ovarian cells with the ability to cross tissue barriers.

### The uPAR_84-95_ sequence promotes dissemination of ovarian CHO-K1 cells to mouse peritoneum

**Figure 7:**
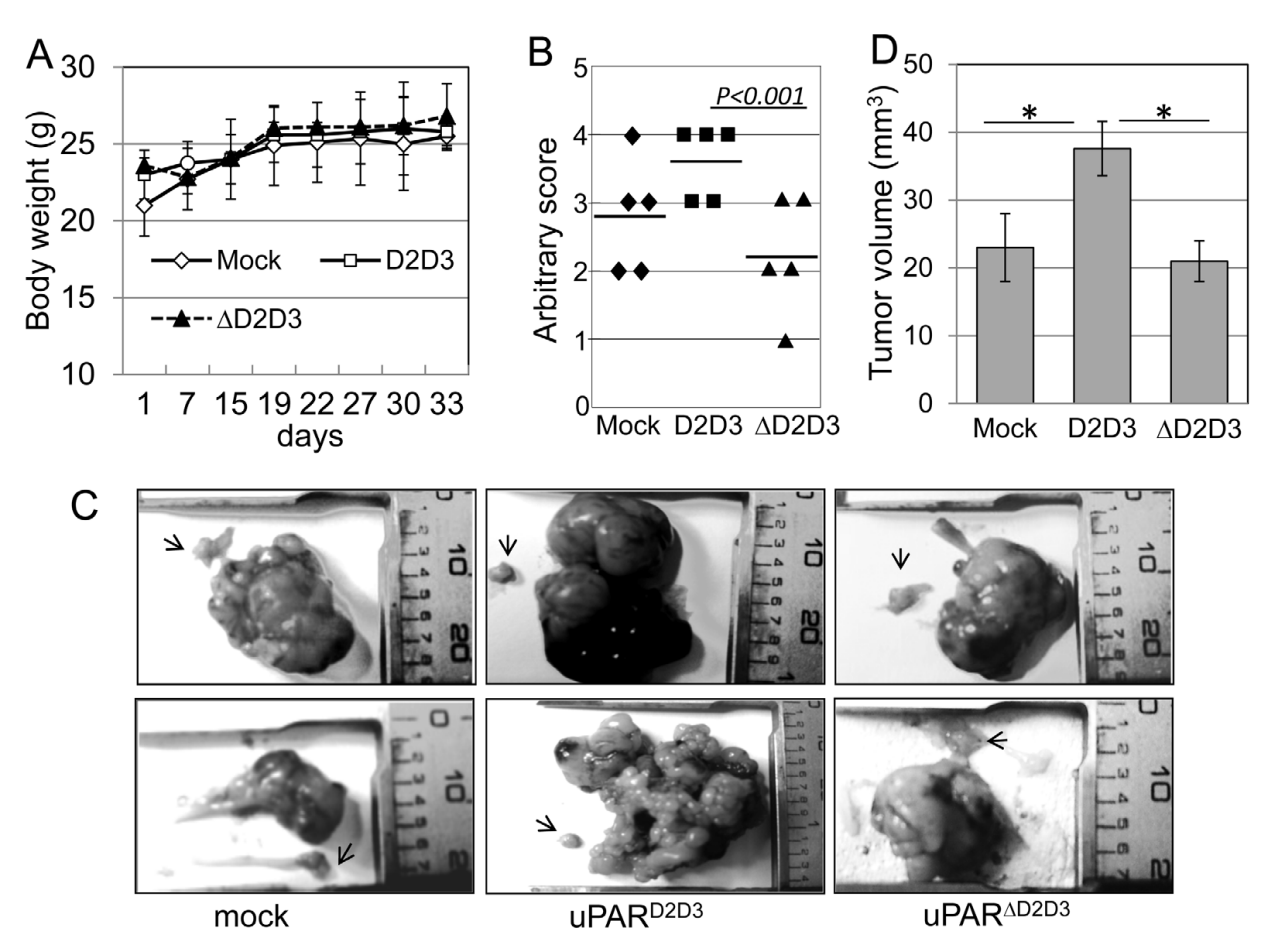
Role of the uPAR_84-95_ sequence in growth and dissemination to mouse peritoneum of ovarian cancer cells Stably transfected CHO-K1 cells (5×10^4^cells/mouse) were orthotopically implanted in 15 Fox n1nu/nu female nude mice (5 mice/group) After 33 days, all mice were sacrificed and the peritoneal cavity carefully inspected for the presence of tumor nodules by two independent observers. **A**. Time-dependent increase in body weight of mice, reported as average of the observed values. **B**. Abdominal dissemination of CHO-K1 transfected cells. Quantification of tumor dissemination in the peritoneal cavity was scored using an arbitrary scale based on the number of the visible tumor nodules in the peritoneal surface and abdominal structures of each mouse: from 0 to +4 (0, for tumor free; +1, for 0-5 nodules/examined organ; +2, for 5-10 nodules/organ; +3, for 10-15 nodules; +4, for >20 nodules including nodular plaques and omental caking). Plots represent data from a total of 5 mice/group. The median values are indicated. **C**. Macroscopic views of ovaries from 2 of the 5 mice injected with CHO-K1/mock, CHO-K1/D2D3 or CHO-K1/ΔD2D3 transfectants. Arrows: control left ovaries. **D**. Tumor volume was measured with a caliper, using the formula: ½ × (width)2 × length. Each bar represents the mean ± SD (n = 5 for each group). *p< 0.05.

Finally, to investigate the ability of the uPAR_84-95_ sequence to favor abdominal dissemination of ovarian cells, CHO-K1/mock, CHO-K1/D2D3 and CHO-K1/ΔD2D3 cells (5×10^4^ cells/mouse) were orthotopically implanted in 15 Fox n1nu/nu female nude mice (5 mice/group). Mice weight was monitored and time-dependent average weight increase showed that all animals survived without changes in body weight (Figure 7A). After 33 days, all mice examined had excess fluid in the peritoneal cavity ranging from 0.25 to 3.0 mL in volume. Therefore, all mice were sacrificed, ascitic fluid discarded and the peritoneal cavity carefully inspected for the presence of tumor nodules. To quantify dissemination of tumor nodules in the peritoneal cavity, we adopted an arbitrary scoring system [8], considering the number of the visible tumor nodules in each mouse, as described in the Materials and Methods. The average score/animal and the mean score of animals in each cohort were calculated. There was no difference in the distribution of metastatic lesions between mice injected with CHO-K1 cells bearing empty vector, D2D3 or ΔD2D3 cDNAs. In contrast, the number of tumor nodules in the peritoneal cavity was significantly increased in mice injected with CHO-K1/D2D3 cells, as compared to CHO-K1/mock or CHO-K1/ΔD2D3 cells (Figure 7B). All mice developed ovarian tumors. Right ovaries were grossly enlarged and tumor size varied from 1.0 to 3.5 cm in diameter, whereas the control ovaries (arrows) looked normal at ~2 mm in diameter (Figure 7C). Histologic analysis of ovarian tumors revealed the presence of large nodules with compact proliferation areas. Residual ovarian tissues of host (eg .oocytes and ovarian surface epithelium) were sporadically observed confined to the periphery of the tumor mass. Although all samples exhibited some necrosis mostly localized in tumor nodules, measurement of necrotic areas revealed not statistically significant differences between D2D3 and ΔD2D3 expressing tumors. Measurement of primary tumor volume (Figure 7D) showed that the kinetics of tumor growth in mice injected with CHO-K1/D2D3 cells (37,6 +/− 4 mm^3^) were significantly faster than those observed in mice injected with CHO-K1/mock or CHO-K1/ΔD2D3 cells (23 +/− 5 mm^3^ and 21 +/− 3 mm^3^, respectively). In addition, ovarian tumors and intestine from mice injected with CHO-K1/D2D3 cells often appeared stuck together (lower panel of Figure 7C).

**Figure 8:**
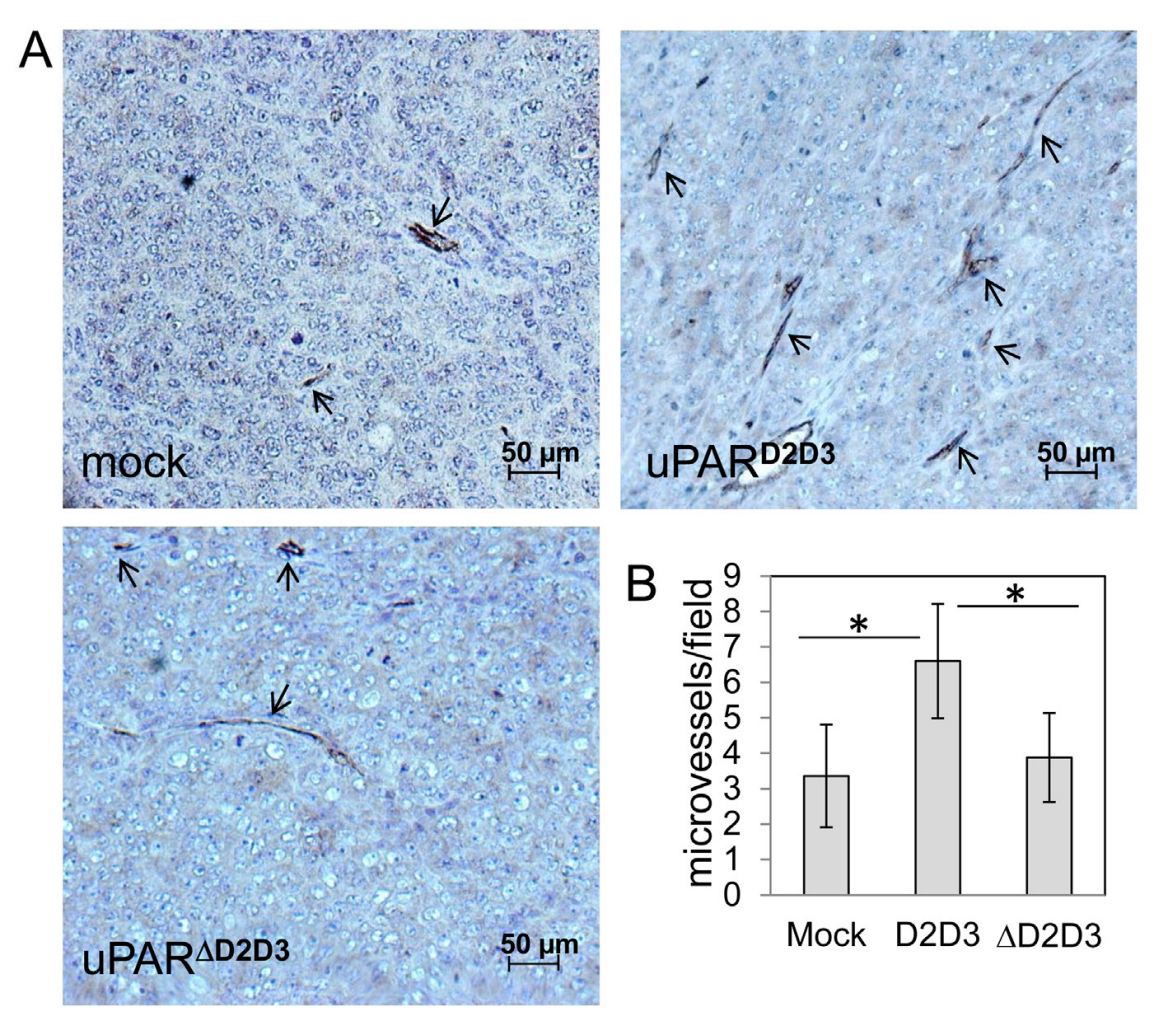
Vascularization of ovarian tumors obtained by uPAR-transfected CHO-K1 cells A Representative images from sections of primary ovarian tumors generated by CHO-K1/mock, CHO-K1/D2D3 or CHO-K1/ΔD2D3 cells. Tumors were excised, fixed in buffered formalin, processed for paraffin sectioning and immune-stained with CD-31 to visualize vascular channels harboring red blood cells (arrows). Scale bar: 50 μm. Original magnification: x200. **B**. Tumor vascularization of primary tumour nodules. Vessels were counted in 5 randomly chosen fields in two CD-31 immuno-stained sections at × 200 magnification on two CD-31 immuno-stained sections in 5 randomly chosen fields per section, in at least two sections per tumor at × 200 magnification. *p< 0.001.

We reasoned that difference in tumor size may not be ascribed to a directed D2D3-dependent cell proliferation increase as all transfected CHO-K1 cells exhibit a comparable proliferation rate in culture (Figure 4E). A possible explanation may be provided by our previous data showing that the chemotactic sequence of uPAR triggers angiogenesis *in vitro* and *in vivo* [29]. Therefore, diversity of tumor size may result from differences in tumor vascularization which, in turn, modulates tumor growth. According to this hypothesis, we noted that ovarian tumors derived from mice in which CHO-K1/D2D3 cells have been orthotopically implanted, appeared more hemorrhagic as compared with ovarian tumors explanted from mice injected with CHO-K1/mock or CHO-K1/ΔD2D3 cells (Figure 7C). This observation encouraged us to analyze intra-tumoral vascularization. Ovarian tumors were fixed in buffered formalin processed for paraffin sectioning, and vascular channels harboring red blood cells were counted in 5 randomly chosen fields in at least two CD-31 immuno-stained sections at × 200 magnification. As shown in the Figure 8 A-B, micro-vessel density increased more than 50% in tumors bearing D2D3 as compared to tumors generated by mock or ΔD2D3 transfected CHO-K1 cells. These results indicate that, in ovarian tumor cells, the uPAR_84-95_ sequence plays a pivotal role in the growth and abdominal dissemination of ovarian cancer cells.

## DISCUSSION

Due to its ability to focus urokinase-dependent proteolytic activity at the leading edge of cancer cells, uPAR has been documented to be involved in the multiple steps of peritoneal metastatic cascade and to play a significant role in ovarian cancer cell-stromal cross-talk [7]. However, to date, the exact mechanistic role of uPAR in ovarian cancer progression and in the development of peritoneal implants has not been shown. The main focus of this study was to elucidate the uPAR role in the intra-peritoneal dissemination of ovarian cancer cells, focusing on the function of the uPAR_84-95_ sequence.

Human peritoneum includes both mesothelial cells and the sub-mesothelial ECM [40]. Thus, metastasizing ovarian cancer cells have two main options for attachment to the peritoneum: mesothelial cells or the exposed ECM. Development of experimental models to analyze this unique mechanism of metastasis represents a remarkable scientific challenge, and many approaches used to study other solid tumors are not transferable to EOC research given the distinct metastatic pattern. Thus, in order to recapitulate key events in EOC metastasis, including invasion of ECM, peritoneum and endothelium, thereby re-establishing morphological and functional features of the corresponding tissue *in vivo*, we have analyzed the ability of ovarian cancer cells to invade a reconstituted basement membrane, mesothelial and endothelial monolayers using a novel technology that records in real-time impedance changes proportional to the number of invading cells. This model may allow to monitor the early events in peritoneal anchoring and invasion of tumor cells and could represent a suitable model to test potential anti-invasive therapeutics.

Herein, we provide evidence that uPAR expressing human ovarian cancer SKOV-3 cells are able to cross matrigel and interrupt monolayer integrity of mesothelial cells, the effects being reduced by antibodies recognizing the uPAR_84-95_ sequence or by the RERF peptide, which specifically inhibits uPAR_84-95_-triggered, FPR-mediated signals. Thus, the uPAR_84-95_ region is likely to be involved in these steps.

uPAR engagement with uPA favors the exposure of the uPAR_84-95_ sequence which, in turn, promotes cytoskeletal rearrangements and directional cell migration by binding to FPR [19]. Since uPA engagement has been proved to force the structure of uPAR to a closed, active conformation in which the 84-95 sequence appears mostly exposed to interactors [26, 41], this finding highlights the role of uPAR but does not identify the specific role of its 84-95 sequence. Moreover, cell-surface cleaved uPAR forms, exposing this sequence at the N-terminus, have been described [28].

To better elucidate the role of uPAR_84-95_ region, we transfected uPAR-negative CHO-K1 cells with cDNAs encoding truncated forms of uPAR, lacking D1 domain and exposing (D2D3) or lacking (ΔD2D3) the 84–95 sequence, or with full length uPAR. CHO-K1/D2D3 cells migrated to a similar extent as the cells transfected with full-length uPAR, unlike CHO-K1/ΔD2D3, which showed a migratory efficiency similar to that of uPAR-negative control cells. D1 removal reduced cell capability to invade matrigel, but CHO-K1/D2D3 were still able to cross matrigel. Accordingly, CHO-K1/D2D3 cells interrupted mesothelial monolayer more efficiently than CHO-K1/ΔD2D3 cells which showed a behavior very similar to the uPAR-negative control cells.

We found that CHO-K1/D1D2D3 cells exhibit the highest capability to invade matrigel, possibly because they retain the ability to bind uPA and focus proteolytic activity on cell surface. Upon D1 proteolytic cleavage, the remaining membrane associated D2D3 domains neither supports uPA-activity at the cell surface, nor binding to vitronectin [18, 28, 42]. However, uPARD2D3 is still able to support an invasive phenotype through its 84–95 sequence, ability that is probably due to its interaction with FPRs; in fact, uPAR and cleaved uPAR appear to mediate different signaling pathways [43]. Furthermore, the integrin αvβ3 has been found to be indispensable for ovarian cancer cell adhesion to mesothelial cells [44], and uPAR interaction with FPR triggers integrin αvβ3 activation. In this scenario, upon enzymatic cleavage of D1 domain, the uPAR_84-95_ sequence could result in a constitutively active uPAR which bypasses the regulatory role of uPA and becomes able to interact with FPR which, in turn, triggers αvβ3 integrin activation.

The peritoneal cavity is particularly receptive to metastasis and ovarian cancer cells can also evade through the peritoneal lymphatics [45]. We found that CHO-K1/D2D3 cells interrupt the integrity of an endothelial monolayer more efficiently than CHO-K1/ΔD2D3 cells, suggesting that uPAR_84-95_ sequence enables ovarian cancer cells to cross tissue barriers and even, intravasate.

CHO-K1 cells transfected with cleaved uPAR forms were also orthotopically implanted in female nude mice; the number of tumor nodules generated by CHO-K1/D2D3 cell spreading to peritoneal cavity was significantly higher as compared to CHO-K1/ΔD2D3 cells, in agreement with the observation that they are endowed with a different invasive potential *in vitro*. Interestingly, we found that tumor volumes were increased in the presence of D2D3 as compared to tumors bearing ΔD2D3. This finding is apparently contrasting with the comparable proliferation rates exhibited by CHO-K1 transfectants. Remarkably, intra-tumor vascularization is enhanced in the presence of uPAR_84-95_ sequence on the surface of CHO-K1 transfectants (Figure 8). In view of the previously reported ability of soluble uPAR_84-95_ to promote *in vitro* and *in vivo* angiogenesis [29], the increased tumor mass could be dependent on the increased tumor vascularization. It is also possible that SRSRY-containing soluble uPAR, shed from CHO-K1/D2D3 cell surface, may enrich the cancer microenvironment with a strong pro-angiogenic factor.

To date, most therapeutics strategies targeting the uPA system by inhibitors of either the uPA-uPAR interaction or uPA proteolysis, have not shown robust anti-tumor activity [46]. However, there is now mounting evidence that uPAR participates to a complex signaling network that control cancer progression, providing a basis for the development of new therapies targeting uPAR interactors [32, 47]. A possibility is to interfere with uPAR/FPRs interaction. Recently, Rea and co-workers described small molecules that impaired cell migration in virtue of their ability to target the Ser ^88^ and Arg^91^residues of uPAR and, consequently, uPAR/FPR interaction [48]. In this context, RERF peptide which, able to interfere with the invasive phenotype of ovarian cancer cells by inhibiting uPAR_84-95_-triggered, FPR-mediated signals, could be considered a valid prototype for the development of new anti-neoplastic therapies designed to simultaneously counteract growth and abdominal dissemination of ovarian cancer cells.

## MATERIALS AND METHODS

### Peptides

Peptides were synthesized by the solid-phase approach and purified by IRBM Science Park, (Pomezia, Italy) that verified their correct sequence by mass spectrometry.

### Cell Culture

Human ovarian carcinoma SKOV-3 and Chinese Hamster Ovary cells - subclone K1 (CHO-K1) cell lines were obtained from the BioBank and Cell Factory of National Cancer Institute of Genova, Italy. SKOV-3 and CHO-K1 cells were cultured in DMEM or F-12 medium, respectively, supplemented with 10% heat-inactivated fetal bovine serum (FBS), penicillin (100 μg/mL), streptomycin (100 U/ml) and maintained at 37 °C in a humidified atmosphere of 5% CO_2_. Human Umbilical Vein Endothelial Cells (HUVEC), purchased by Lonza, were employed between the third and the seventh passage according to Arnaoutova et al. [49], and grown in Eagle Basal Medium supplemented with 4% FBS, 0.1% gentamicin, 1μg/mL hydrocortisone, 10 μg/mL epidermal growth factor and 12 μg/mL bovine brain extract (Cambrex).

### Primary culture of human peritoneal mesothelial cells

Human peritoneal mesothelial cells (HPMC)s were purified and characterized according to Stylianou et al. [35, 50]. Briefly, specimens of human omentum were obtained from consenting patients undergoing elective abdominal surgery (~2 cm^2^). Blunt dissection removed excess fat and provided predominantly transparent samples of tissue. The omentum was washed several times with sterile phosphate buffered saline (PBS) to remove any contaminating red blood cells. Omental specimens were subjected to disaggregation with 5 mL of 0.125% (wt/vol) trypsin, 0.01% (wt/vol) EDTA (Sigma) for 20 min at 37°C with continuous rotation. Then, the suspension was centrifuged at 800 × g for 5 min, the cell pellet was washed once in F12 culture medium containing 10% (vol/vol) FBS, suspended in the same medium to a volume of 5 mL and seeded in 25 cm^2^ tissue culture flasks. Half the medium was exchanged 24 hrs later and fully replaced once every three days. The mesothelial phenotype was identified by the uniform cobblestone appearance at confluence, by the lack of staining for factor VIII-related antigen, and by the positive staining for cytokeratins 8 and 18 and vimentin. To obtain a monolayer of HPMCs 1 × 10^3^ cells, employed between the second to the third passage, were seeded on E-16-well plates having the same volume of 96-well plates.

### Plasmids and transfections

The expression vector pcDNA3-uPAR (D1D2D3) was constructed by inserting the 1027 bp EcoRI-EcoRI fragment from pBluescript II SK, containing the whole human uPAR-cDNA [39]. uPAR cDNAs lacking the N-terminal D1 domain and exposing (D2D3) or lacking (ΔD2D3) the 84–95 sequence were generated as previously described [18]. All sequences were confirmed by DNA sequencing. The expression vectors pcDNA3-uPARD1D2D3, pcDNA3-uPARD2D3 and uPAR ΔD2D3 were transfected into cells using FuGENE 6 transfection reagent, according to the manufacturer's specifications (Roche Applied Science). Transfected cells were selected by Geneticin at 0.8 mg/mL for 15 days, pooled and cultured in the presence of 0.5 mg/mL Geneticin.

### Flow cytometry

Detached cells (0.5×10^6^ cells/sample) were incubated with PBS, non-immune serum or 2 μg/ml R4 anti-uPAR monoclonal antibody for 30 minutes at 4°C. After extensive washing with PBS, cells were incubated with 1: 200 Alexa 488-conjugated F(ab')2 fragment of rabbit anti-mouse IgG (Molecular Probes) for 30 min in the dark and, finally, re-suspended in 0.5 mL PBS. Samples were analyzed by flow cytometry using a FACS Aria II and DIVA software (Becton & Dickinson).

### Western blot

Cells detached using 200 mg/L EDTA, 500 mg/L trypsin (Cambrex), were lysed in RIPA buffer (10 mM Tris pH 7.5, 140 mM NaCl, 0.1 %SDS, 1% Triton X-100, 0.5% NP40) containing protease inhibitor mixture. Protein content of cell lysates was measured by a colorimetric assay (BioRad). Twenty-five nanograms of proteins were separated on 10% SDS-PAGE and transferred onto a polyvinylidene fluoride membrane. The membranes were blocked with 5% non-fat dry milk and probed with 1 μg/mL R4 anti-uPAR monoclonal antibody recognizing uPAR D3 domain, or 1 μg/mL α-tubulin polyclonal antibody (Life Technologies). Washed filters were incubated with horseradish peroxidase-conjugated anti-mouse or anti-rabbit antibody and detected by ECL (Amersham). Densitometry was performed by NIH Image 1.62 software (Bethesda, MD).

### Cell proliferation

Cell proliferation was assessed using E-16-well plates and the xCELLigence technology (Acea Bioscience, distributed by Roche Diagnostics) as described [51]. Briefly, cells (2×10^3^/well) were seeded in 16-well plates in growth medium and left to growth for 72 hrs. Microelectrodes placed on the bottom of plates, detect impedance changes which are proportional to the number of adherent cells and are expressed as Cell Index. The impedance value of each well was automatically monitored by the xCELLigence system and expressed as a Cell Index value. Doubling times for each cell clone were calculated from the cell growth curve during the exponential growth. The experiments were performed at least twice in quadruplicate.

### Fluorescence microscopy

Cells (~2×10^4^/sample) were seeded on glass coverslips and cultured for 24 hrs in growth medium. Then, slides were washed with PBS, fixed with 2.5% formaldehyde in PBS for 10 min at 4°C and incubated overnight at 4°C with 2 μg/mL R4 anti-uPAR monoclonal antibody or rabbit anti-uPAR_84-95_ polyclonal antibody [30]. HPMCs plated on glass slides (30%-40% confluence), were fixed and permeabilized with 2.5% formaldeyde-0.2% Triton X-100 in PBS for 10 min at 4°C, then incubated with 2 μg/mL anti-vimentin monoclonal antibody (Dako), anti-cytokeratin 8/18 polyclonal antibody (MyBiosurce), or anti-von Willebrand factor VIII monoclonal antibody (Dako) for 1 hr at 4°C. Then, 1:800 goat Alexa Fluor 488 anti-rabbit IgG, Alexa Fluor 488-conjugated F(ab')2 fragment of rabbit anti-mouse IgG, or Alexa Fluor 594 goat anti-mouse IgG (Molecular Probes) were applied to slides at 23°C for 40 minutes. After nuclear staining with 4-6-diamidino-2-phenylindole dye (DAPI), coverslips were mounted using 20% (w/v) mowiol and analyzed by a fluorescence inverted microscope connected to a video-camera (Carl Zeiss). To analyze FPR internalization, cells grown on glass slides were exposed to 10 nM N-formyl-Nle-or Leu-Phe-Nle-Tyr-Lys-fluorescein (Molecular Probes), diluted in serum-free DMEM for 30 minutes at 37°C as described [32].

### Cell migration assays

Chemotaxis assays were performed in Boyden chambers, using vitronectin-coated 8μm pore size PVPF-filters (Nucleopore) as previously described [30]. Briefly, 1×10^5^ viable cells were seeded in each upper chamber in serum-free medium. The lower chamber was filled with serum-free medium containing 10 nM fMLF, 10 nM SRSRY peptide or 10% FBS as chemoattractants. Cells were allowed to migrate for 4 hrs at 37°C, 5% CO_2_. At the end of the assay, cells on the lower filter surface were fixed with ethanol, stained with haematoxylin and 10 random fields/filter were counted at 200x magnification. The arbitrary value of 100% was given to the basal cell migration, assessed in the absence of chemoattractant. All experiments were performed at least twice in triplicate, and the results were expressed as percentage of the basal cell migration.

### Matrigel invasion by ovarian cells monitored by the xCELLigence RTCA system

Matrigel invasion assays were performed using CIM-16-well plates and the xCELLigence RTCA technology as described [51]. CIM plates are provided with interdigitated gold microelectrodes on bottom side of a filter membrane which is interposed between a lower and an upper compartment. Filters were coated with 50 μg/well matrigel diluted in serum free medium. The lower chamber was filled with growth medium. Ovarian cells (2×10^4^ cells/well) were seeded on filters in serum-free DMEM. Microelectrodes detect impedance changes which are proportional to the number of cells invading matrigel and are expressed as Cell Index. Invasion was monitored in real-time for 18 hrs. Each experiment was performed at least twice in quadruplicate.

### HPMC and HUVEC invasion by ovarian cells monitored by the xCELLigence RTCA system

These assays were performed using E-16-well plates. HPMCs (5×10^3^ cells/well) or HUVECs (1×10^4^ cells/well) suspended in growth medium, were seeded in E-16-well plates and allowed to adhere for 4 hrs (HPMC) or grow for 20 hrs (HUVEC) until they form a confluent monolayer, prior to seeding ovarian cells (2×10^4^ cells/well) in the presence of 10% FBS plus/minus 10 nM indicated peptides, 2 μg/mL normal rabbit serum on (NRS), purchased by Millipore or 2 μg/mL rabbit anti-uPAR_84-95_ polyclonal antibody. When HPMCs or HUVECs are challenged with invading cells, there is a drop in electrical resistance within 2-8 hrs which is monitored in real-time as the Cell Index changes due to invasion of the mesothelial or endothelial monolayer. The experiments were performed at least twice in quadruplicate.

### Orthotopically implantation of ovarian cells in nude mice

Fifteen six-eight week old (21 to 23 g), Foxn1nu/nu female nude mice (Harlan), were maintained in a germ-free environment. Housing and handling of mice were in accordance with institutional guidelines complying with national and international laws and policies. Transfected CHO-K1/mock, CHO-K1/D2D3 or CHO-K1/ΔD2D3 cells were orthotopically implanted in nude mice. Specifically, mice were anesthetized and subjected to a small dorsal incision. Right ovaries were externalized prior to the injection of cells (5×10^4^cells/mouse as a single-cell suspension in 30μl of sterile PBS, 98% viability) under the bursal membrane, using a 30-gauge needle. After surgery, mice were monitored daily and time-dependent average weight was monitored every three-seven days for 33 days. At this time, mice were sacrificed by cervical dislocation and the peritoneal cavity carefully inspected for the presence of cancer cell dissemination. According to Al-Hassan et al., [8], tumor dissemination in the peritoneal cavity was scored on an arbitrary scale based on the number of tumor nodules in the peritoneal surface and abdominal structures from 0 to +4 (0, for tumor free; +1, for 0-5 nodules/examined organ; +2, for 5-10 nodules/organ; +3, for 10-15 nodules; +4, for >20 nodules including nodular plaques and omental caking). The length and the width of the primary tumors were measured with the help of a caliper and the volume was calculated using the formula: ½ × (width)2 × length (mm). Excised tumors were fixed in buffered formalin and processed for paraffin sectioning. Tumor vascularization was assessed by counting vascular channels harboring red blood cells on CD31 [52] immuno-stained sections in 5 randomly chosen fields per section, in at least two sections per tumor at × 200 magnification.

### Statistical analysis

The results are expressed as the means ± SD of the number of the indicated determinations. Data were analyzed by one-way analysis of variance and post hoc Bonferonni's modified t-test for multiple comparisons.

### Ethics statement

The research work with mouse model has been approved by Institutional Ethical Committee of Istituto Nazionale Tumori “Fondazione G. Pascale”-IRCCS, Naples, Italy (protocol n. 09, December 20th, 2010).
